# Executive functions and mathematical competence in Chinese preschool children: A meta-analysis and review

**DOI:** 10.3389/fpsyg.2022.1012660

**Published:** 2022-11-18

**Authors:** Zhiyong Zhong, Yongqi Xu, Ruining Jin, Chen Ye, Mengmeng Zhang, Hao Zhang

**Affiliations:** ^1^School of Education, Minzu University of China, Beijing, China; ^2^Civil, Commercial and Economic Law School, China University of Political Science and Law, Beijing, China; ^3^Department of Preschool Education, Beijing Union University, Beijing, China; ^4^School of Foreign Languages, China University of Petroleum, Beijing, China

**Keywords:** executive functions, mathematical competence, meta-analysis, China, preschooler

## Abstract

Numerous studies have been conducted on the correlation between preschool children’s executive functions and mathematical competence, the findings of those studies are inconsistent. This study used meta-analysis to investigate the correlation between executive functions and mathematical competence of preschool children in China, and then explored the moderating effects of age, gender, and instruments. The researchers conducted an extensive search of CNKI, Web of Science, Google Scholar and used screening criteria to identify 22 studies of Chinese preschoolers aged 3–7 years from 2007 to 2021. The findings indicated that there was a positive correlation between Chinese preschool children’s executive functions and mathematical competence (*r* = 0496), of which the effect size between mathematical competence and inhibition control was 0.347, and the effect size for working memory was 0.432, with an effect size of 0.370 for cognitive flexibility. Furthermore, Moderation analysis suggests that the preschoolers’ age, gender, and instruments affected the association between the executive functions and mathematical competence. The implications of these findings for theory and practice deserve further discussion.

## Introduction

Mathematical competence is a very important basic ability in modern society, and it is also a vital component of academic competency and development foundation, particularly in preschool and school-age children (Campbell, 1989; Bull et al., 2008; Jordan et al., 2022). For many years, researchers have attempted to solve mysteries behind the complex process of various children’s mathematical competence for further study, which would help better understand the influence factors of children’s mathematical competence. Executive functions, on the other hand, constitute one of the cognitive processes directly tied to children’s mathematical competence and have aroused a great deal of attention (Jordan et al., 2012; Zeng, 2020; Zhang, 2021).

Executive functions are also considered a critical advanced cognitive activity that influences individual development (Zelazo et al., 2004; Anderson and Reidy, 2012; Vanhala et al., 2022). In other words, children’s executive functions are formed and continually developed from the earliest stage of infancy till puberty. Therefore, the development of executive functions would affect not just children’s daily lives, but also their academic achievement once they start their school lives. It is noted that executive functions have multiple sub-dimensions, which makes studies on executive functions more challenging because different executive functions may lead to varied consequences on mathematical competence (Oeri et al., 2020).

In China, influenced by traditional values which focus on the cultivation of children’s competitiveness at an early age, parents tend to place a high value on the development of children’s mathematical competence in the preschool stage (Luo and Yang, 2016; Sun et al., 2022). Some parents, for instance, expect to improve their children’s mathematical competence through executive functions training (Zeng, 2020). However, whether executive functions can effectively promote the improvement of preschool children’s mathematical competence is debatable, as there is an inconsistency with the current research conclusions in the Chinese context. Namely, existing empirical studies have found that the correlation coefficient range of executive functions’ influence on children’s mathematical competence is significantly different between 0.01 and 0.74, (e.g., Liu, 2013; Xiao et al., 2015; Wang, 2019), therefore it is worthwhile to further clarify the prediction effect between the two variables by means of meta-analyses.

For this purpose, we employed a meta-analytic approach to synthesize the extant literature examining the interrelations between executive functions and preschool children’s mathematical competence in China. We also examined whether the associations between executive functions and preschool children’s mathematical competence varied as a result of some study moderators. Specifically, we addressed two research questions: (1) What is the overall strength of the relationship between executive functions and preschool math competence when the study conceptualizes executive functions as a multidimensional structure? (2) Does the direction and strength of the association between executive functions and preschool math competence differ on account of the child’s gender, grade level, age, or the measurement method of executive functions?

### Domains of executive functions

According to Baddeley and DellaSala (1996), executive functions are defined as the generality of controlling and coordinating various cognitive processes to ensure that the cognitive system achieves specific goals in a flexible and optimized manner when performing complex cognitive tasks, and the essence of the control mechanism is to regulate other cognitive processes for the fundamental purpose of producing coordinated, orderly and purposeful behaviors. Simply put, the executive function is a higher-level cognitive function as a cognitive processing mechanism that consciously guides targeted behaviors. Li and Wang (2004) divided executive functions into cold executive functions and hot executive functions from the functional perspective. The division is based on whether there is emotional involvement in the cognitive process. A classic paradigm of hot executive functions is delayed gratification, which examines whether children can resist temptation and wait a little longer for more rewards. In the current study of the association between executive functions and mathematical competence, children did not need to resist any temptation, so this study only considered children’s cold executive functions.

In recent years, there have been some differences in the division of the core elements of executive functions. While Baddeley (1996) divided executive functions into four subcomponents, i.e., inhibition control, working memory, cognitive flexibility, and dual-task coordination, others contend that there are three core components: inhibition control, working memory (updating), and cognitive flexibility (shifting) (Miyake et al., 2000; Diamond, 2013; Friedman and Miyake, 2017). Although the differences regarding the categorizations of components of executive functions are controversial, in essence, working memory and updating, cognitive flexibility, and shifting have a similar connotation. Since most previous studies regarding executive functions used the three core components categorization, in this study, we will also use the three core components and discuss inhibition control, working memory, and cognitive flexibility.

Perner and Wimmer (1985) pointed out that inhibition control mainly inhibits interfering stimuli, and the individual realizes the overall cognition and representation of the target by realizing the exclusion of interfering stimuli. Aydmune et al. (2017) defines inhibition control as a spontaneous cognitive behavior and emphasizes the important role of inhibition control in the complex cognitive process of cognitive goals. Other researchers believe that inhibition control is produced spontaneously by the individual, and what the behavior actively inhibits is not the irrelevant interfering stimulus, but the individual’s spontaneous dominant response (Miyake et al., 2000; Zeng, 2020). According to different classification standards, inhibition control is also divided into different types. For example, Harnishfeger and Bjorklund (1994) proposed that inhibition control could be divided into conscious control and unconscious control, depending on whether it occurs automatically. Conscious inhibition occurs automatically, and unconscious inhibition occurs when a stimulus is activated. Inhibition will be implemented when it is identified as disturbing information. Based on the stage of inhibition, inhibition control can also be divided into cognitive inhibition and behavioral inhibition (Li et al., 2021). Cognitive inhibition occurs in the process of psychological activities, while behavioral inhibition is the impulse or activity response to inhibiting behaviors (Everett and Lajeunesse, 2000). Baddeley (2013) also pointed out that different from short-term memory, working memory could store and process information at the same time, promoting a series of cognitive activities, such as imagery, speech, planning, learning, problem-solving, and decision-making. According to Per Kane et al. (2001), working memory is a kind of executive attention that when external stimuli and background information enter the cognitive process, the individual can eliminate interference, effectively suppress information and responses irrelevant to cognitive goals, and can maintain long-term active retrieval and retention of information in memory. Martin and Rubin (1995), on the other hand, shed light on the definition of cognitive flexibility, defining it as the ability to maneuver to adapt to the environment, which includes two aspects: first, the individual’s awareness of autonomy and flexible choices and the ability to actively adapt to the environment to complete a cognitive task, as well as the belief in one’s own ability to adapt; second, the cognitive process of modifying cognitive processing strategies to adapt to the environment. In addition, Miyake and Friedman (2012) proposed that the development of cognitive flexibility is based on working memory and inhibition control and is influenced by the development of the brain’s prefrontal lobes. Taken together, the researchers think that executive functions are comprised of three key components, i.e., inhibition control, working memory, and cognitive flexibility.

### Domains of mathematical competence

On the one hand, the National Council of Teachers of Mathematics (NCTM) divided children’s mathematical competence into the ability to master mathematical learning content and the ability shown in the process of mathematical learning in an American Context (National Council of Teachers of Mathematics, 1989). Specifically, the ability to master mathematical learning content means the ability of children to master basic mathematical learning content such as counting, simple symbol representation, and simple operation, while the ability shown in the process of mathematical learning denotes the ability of children to use mathematical knowledge to solve problems encountered in life based on understanding basic mathematical knowledge, and further carry out mathematical learning (Graf, 2009). On the other hand, although there is no clear academic definition of preschool children’s mathematical competence in China, *The Guidelines on Learning and Development for Children Aged 3–6 (2012)* issued by the Ministry of Education of the People’s Republic of China set two clear goals for mathematical cognitive development from an educational perspective: to preliminarily perceive the usefulness and fun of mathematics in life and to perceive and understand quantities and relations between and among quantities.

In terms of mathematical competence, Luo and Yang (2016) detailed preschool children’s mathematical competence into four basic abilities: reading and writing numbers, mastering number facts, acquiring arithmetic skills, and understanding mathematical concepts. Aside from abilities, mathematical competence is also seen as an intrapersonal characteristic, as some researchers view preschool children’s mathematical competence as a kind of personality and psychological characteristic through successful completion of learning activities and solving mathematical problems (Wen et al., 2007). Based on the differences in acquisitions, the mathematical competence of preschool children is divided into formal mathematical competence and informal mathematical competence (Zhang, 2021). Formal mathematical competence is acquired through systematic training in preschool, including the concept of number symbols, reading, and writing of numbers, addition, subtraction, multiplication and division, and the concept of place value; on the other hand, informal math competence is the math competence children acquire in their daily lives, including counting, comparing numbers, and simple operations.

### The correlation between executive functions and mathematical competence

Some researchers have pointed out that executive functions and mathematical competence may share a common physiological basis, which involves the prefrontal lobe, orbitofrontal lobe, medial thalamic system of the brain, etc. (Xu et al., 2004; He, 2012). However, many of the previous studies found inconsistent or small, or moderate correlations between the coefficients of total executive functions and mathematical competence (Liu, 2013; Xiao et al., 2015; Feng, 2021). These inconsistencies and different effect sizes may be attributable to variations in executive function dimensions or mathematical competence within any specified research. Specifically, in terms of inhibition control, Blair and Peters Razza (2007) sampled 141 children aged 3–5 to test their inhibition ability through the motor inhibition paradigm, and found that there was a significant correlation between inhibition control and mathematical competence; and Miller et al. (2013) found that there was no significant correlation between inhibition control and mathematical competence in preschool children through a study of 129 children aged 3–5. In terms of working memory, there were also inconsistencies in research findings. For example, Bull et al. (2008) and his colleagues indicated that working memory played an important role in the development of preschool children and could significantly predict children’s mathematical achievement. Alloway et al. (2005) found that working memory could not significantly predict the mathematical competence of preschool children. There are also differences of opinions on cognitive flexibility. According to the findings of Espy (1997), cognitive flexibility cannot significantly predict the mathematical competence of preschool children, while Zhou et al. (2009) believe that cognitive flexibility has a significant effect on the mathematical competence of preschool children in the Chinese context. In addition, there are differences of opinions regarding the strength of the correlation between the subcomponents of executive functions and mathematical competence. A cross-sectional study (e. g., Ilona et al., 2013) and a longitudinal study (e. g., Fuchs et al., 2010) showed that working memory was more closely related to children’s mathematical competence, but some studies believed that inhibition control was the most core part of each sub-component of executive functions (Everett and Lajeunesse, 2000; Liu, 2013; Aydmune et al., 2017)). To sum up, there are many inconsistent research results on the association between executive functions and mathematical competence of preschool children. Therefore, a synthesized study on the association between executive functions and preschool children’s mathematical competence is needed for us to further understand how this association differs.

### Potential moderators of the link between executive functions and mathematical competence

There are differences in children’s mathematical cognition of different genders. Studies have shown that gender differences in children’s mathematical development begin to appear at the ages of 5–6 (Jordan et al., 2006; Liu and Tao, 2012). Compared with boys, girls’ mathematical ability is relatively weak, which may be related to expressive ability (Skogli et al., 2013). In addition, there may also be gender differences in the cognitive mechanisms that construct mathematical concepts, as well as differences in brain development and hormone levels (Zhou and Chen, 2011). Thus, gender may moderate the association between executive functions and mathematical competence in preschoolers.

With the growth of children’s age, especially when children enter kindergarten, due to the stimulation and reinforcement of the external environment such as the interaction with their teachers and peers, children’s ability in inhibition control has been continuously strengthened (Li et al., 2021). In addition, as children gradually grow and mature, their brain activation areas related to mathematical activities move from the prefrontal lobe to the parietal region, and children’s memory, perception, spatial abilities, and general cognitive abilities that affect children’s mathematical learning continue to develop (Fei et al., 2020). For older children, parents encourage them to reach out, contact and perceive mathematics in advance through intermediaries such as games and math picture books, thereby accumulating certain mathematics learning experience (He, 2012). Thus, age may moderate the association between executive functions and mathematical competence in preschoolers.

Finally, some studies have implied that instruments may influence the association between executive functions and mathematical competence (Wen et al., 2007; Zhou et al., 2009; Zhang, 2021), since the measurement of executive functions is based mainly on selecting corresponding instruments for different sub-components. For example, the commonly used instruments in inhibition control are Stroop Task, Simon Task, Flanker Task, Negative Priming, etc. The measurement of working memory has a six-box task, dual-task paradigm, N-back paradigm, active memory paradigm, etc. The measures of cognitive flexibility mainly include two types of instruments, namely, Dimensional Change Card Sort (DCCS), and Flexible Induction of Meaning (FIM), etc. The instruments used in diverse studies are different, and researchers often choose the corresponding instruments according to the actual situation. Although some instruments measure the same ability, there are vast variances in the measurement contents. For example, in the measurement of children’s inhibition control, in addition to using the classic paradigm, i.e., Stroop task, to test, there are also studies using listening and seeing tasks and color tasks, which are quite different in content. Therefore, instruments may moderate the association between executive functions and mathematical competence in preschoolers.

### Current study

As shown above, the association between executive functions and mathematical competence remains unclear in Chinese preschool children. What is the real relationship between executive functions and mathematical competence? How do subcomponents of executive functions impact early childhood math abilities? These fundamental problems have not been well resolved. However, as the research shows, the relationship between the two is complex (Sun, 2003; Miller et al., 2013). One is that the two may not be strongly related but may be moderately related. The second is the relationship between the sub-components of executive functions and mathematical competence. The correlation between the two is relatively ambiguous and needs further study. Thirdly, although executive functions (including each of its sub-components) are related to mathematical competence, they may not constitute a direct causal relationship, and may also be influenced by other factors. Based on this, using the meta-analysis method, the current study has two research aims: (1) to determine whether there is a significant correlation between executive functions and mathematical competence in the Chinese context, and (2) to determine whether domains of EF, age, gender, and instruments may moderate their links.

## Materials and methods

### Inclusion criteria

Articles that met the following criteria were included: (a) The research subjects were Chinese preschool children, aged 3–7 years; (b) The study was required to report the difference between executive functions (including inhibition control, working memory, cognitive flexibility, etc.) and mathematics; (c) The research does not include special groups of children, such as children with learning disabilities; (d) The research needs to report basic information such as the gender, age, and instruments of the subjects; and (e) The research also includes master’s and doctoral dissertations.

### Data retrieval strategies

Firstly, through the retrieval of CNKI, Wanfang Data, VIP, Google Scholar, Web of Science, ProQuest, Scopus, etc., with “Executive Functions” “Inhibition control” “Working Memory” “Cognitive Flexibility” and “Mathematics” “Chinese” as keywords, 1,452 search results were initially obtained, and the retrieval time was from December 1, 2021, to February 1, 2022. Secondly, after the titles and abstracts of these literatures were studied, those that did not meet the inclusion criteria were excluded, and the abstracts and full texts of the literatures with potential research value were read. In the end, a total of 22 studies in line with the established research purpose were obtained, involving 2,730 subjects, with a total of 65 effect sizes, and the time span was 2007–2021. The specific screening process is as follows:

### Coding procedure

Each study was coded according to the following characteristics: authors’ information, year of publication, source of subjects, sample size, number of boys and number of girls, their ages, instruments, and correlation coefficients between executive functions and mathematical competence (see Table 1). For the entry of correlation coefficients, if the original literature only reports the correlation coefficients between the sub-dimensions of executive functions and mathematics, it will be calculated according to the formula $r_{xy}=\frac{\Sigma_{i=1}^{m}r_{x_{i}y}}{\sqrt{m+\left(m-1\right)\bar{r}xx}}$ (Pigott, 2006) the correlation coefficient between synthetic executive functions and mathematics will be encoded. The entire coding process was completed independently by two coders, and the coding consistency reached 97.5%. If there is a coding inconsistency, we will return to the original literature for discussion and make corrections in a timely manner.

**Table 1:** Summary of studies included in the meta-analysis.

Reference	City	Population	Males	Females	Age	Variable	Instruments	Correlation coefficient(r)
Liu (2013)	Dalian	49	23	26	4–7 years	EFs/IN/UP/*CF* & MC	Frye D Task	0.016; 0.733; 0.240; 0.521
Zeng (2020)	Changsha	52	26	26	4–6 years	EFs & MC	HTKS task	0.436
Xiao et al. (2015)	Hunan	101	49	52	4–6 years	EFs/IN/UP/*CF* & MC	Stroop task; Digit backwards task; FIST Task	0.22; 0.27; 0.18; −0.01
Fei et al. (2019)	Shenzhen	196	103	93	5–6 years	EFs/IN/UP/*CF* & MC	Stroop Task; Fixed box Task; WCST Task	0.491; 0.319; 0.264; 0.323
Wen et al. (2007)	China	64	30	34	6–7 years	EFs/IN/UP/*CF* & MC	Stroop task; Fixed box task; WCST task	0.53; 0.47; 0.37; 0.39
Cheng et al. (2019)	Shanghai	113	58	55	6–7 years	UP& MC;	Digit backwards Task	0.74
Ren (2015)	Chengdu	92	45	47	4–6 years	EFs/IN/UP/*CF* & MC	See-hear task; Fixed box task; FIST task	0.711; 0.582; 0.588; 0.668
Liu (2019)	Hubei Province	134	78	56	3–6 years	EFs/IN/UP/*CF* & MC	Red-blue Task; Fixed box Task; FIST task	0.72; 0.71; 0.69; 0.46
Hu et al. (2014)	Chongqing	58	26	32	3–6 years	IN/UP& MC	Stroop task; Fixed box task; FIST task	0.498; 0.264
Feng (2021)	China	114	58	56	4–6 years	EFs/IN/UP/*CF* & MC	See-hear task; Fixed box task; Red rabbit blue bear task	0.81; 0.63; 0.74; 0.64
Fei et al. (2020)	Shenzhen	200	94	106	3 years	EFs/IN/UP/*CF* & MC	Stroop task; Fixed box task; WCST task	0.348; 0.301; 0.215; 0.335
Fei et al. (2020)	Shenzhen	210	113	97	4 years	EFs/IN/UP/*CF* & MC	Stroop task; Fixed box task; WCST task	0.335; 0.225; 0.229; 0,269
Xie, (2021)	Shanghai	70	42	28	4–6 years	IN&MC	Stroop task	0.476
Qin (2017)	Shanghai	120	58	62	5–6 years	EFs/IN/UP/*CF* & MC	Smile-cry task; Digit-Span task; FIST task	0.565; 0.348; 0.464; 0.337
Zhang (2021)	Shanghai	269	138	131	3–6 years	EFs & MC	HTKS task	0.67
Zhang, (2018)	Beijing	96	57	39	5 years	EFs/IN/UP/*CF* & MC	NIH toolbox task	0.31; 0.33; 0.28; 0.02
Wang (2019)	Nanjing	59	33	26	4–5 years	EFs/IN/UP/*CF* & MC	Stroop task; Digit backwards task; FIST task	0.624; 0.314; 0.701; 0.317
Fung et al. (2020)	Hong Kong	172	88	84	5–6 years	EFs & MC	HTKS task	0.17
Chung et al. (2017)	Hong Kong	97	49	48	3–4 years	EFs & MC	HTKS task	0.53
Yang et al. (2019)	Hong Kong	165	87	78	4–5 years	EFs/IN/UP/*CF* & MC	Stroop task; Digit-span task; Keep track task	0.40; 0.36; 0.20; 0.32
Georgiou et al. (2020)	China	181	99	82	6–7 years	EFs/IN/UP/*CF* & MC	Expressive attention; Digit backwards task; Planned connections	0.51; 0.31; 0.45; 0.42
Lan et al. (2011)	Beijing	118	59	59	4–5 years	IN/UP&MC	HTKS task; The sentence completion task	0.22; 0.29

EFs, executive functions; IN, inhibition control; UP, working memory; CF, cognitive flexibility; MC, mathematical competence.

### Effect size

In this study, the correlation coefficient between variables was used as an indicator of the effect size. To exclude the influence of the sample size on the effect size results, the study performed Fisher’s Z transformation on the *r* value, that is, Z = 0.5× Log((1 + r)/(1-r)), SE = 1/√((n-3)). As suggested by Cohen et al. (2002), we believe that when the correlation coefficient is less than 0.10, the effect size is small; when the correlation coefficient is 0.30, the effect size is moderate; when the correlation coefficient is greater than 0.5, the effect size is large.

### Statistical analysis

Some studies reported the correlation coefficient between a sub-dimension of executive functions and mathematical competence, some studies reported the correlation coefficient between three sub-dimensions (inhibition, working memory, and cognitive flexibility) and mathematics, and some studies’ correlation coefficients between preschool children’s executive functions overall and mathematical competence were reported. In this study, all the above correlation coefficients were included in the statistics, the Pearson correlation coefficient *r* between variables was used as the effective value for correlation calculation, and the meta-analysis software Comprehensive Meta-Analysis Version 3.3 was used for data processing and analysis. In addition, the study calculated weights and 95% confidence intervals based on the sample size.

## Results

### Publication bias analysis

**Figure 1:**
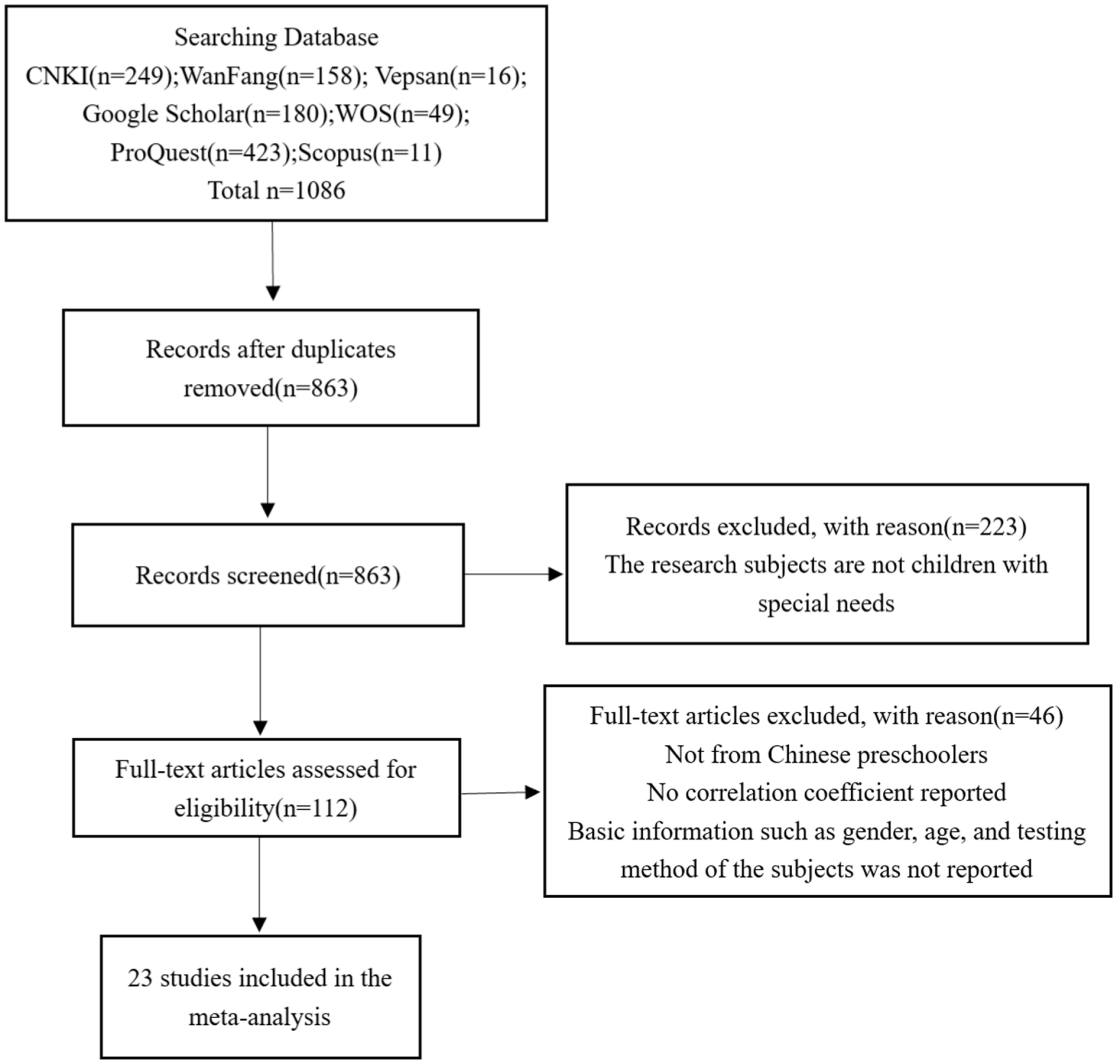
Flowchart of the inclusion protocol.

**Figure 2:**
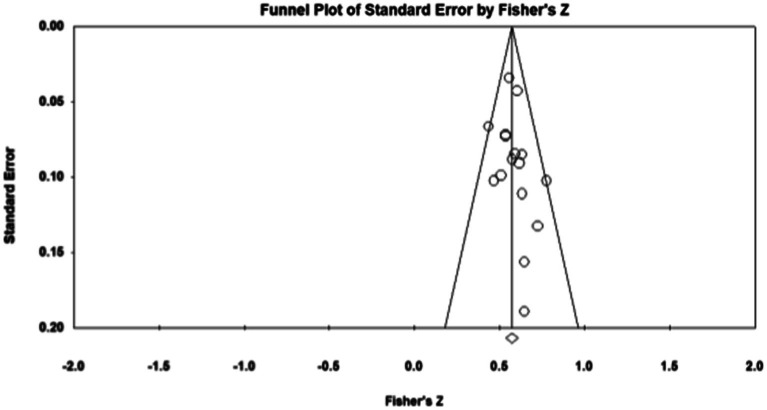
Funnel plot. Executive functions—Mathematics.

**Figure 3:**
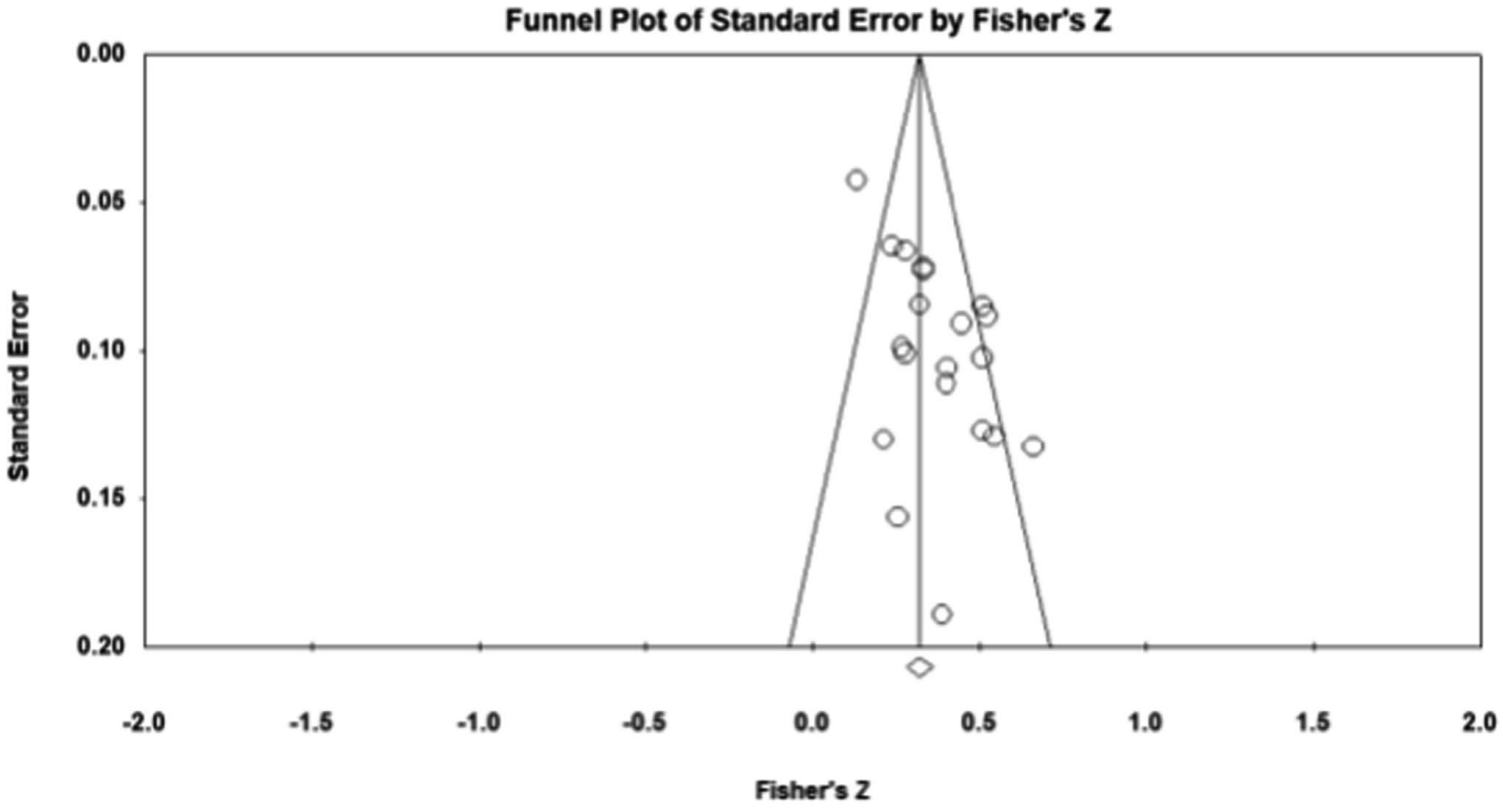
Funnel plot. Inhibition—Mathematics.

**Figure 4:**
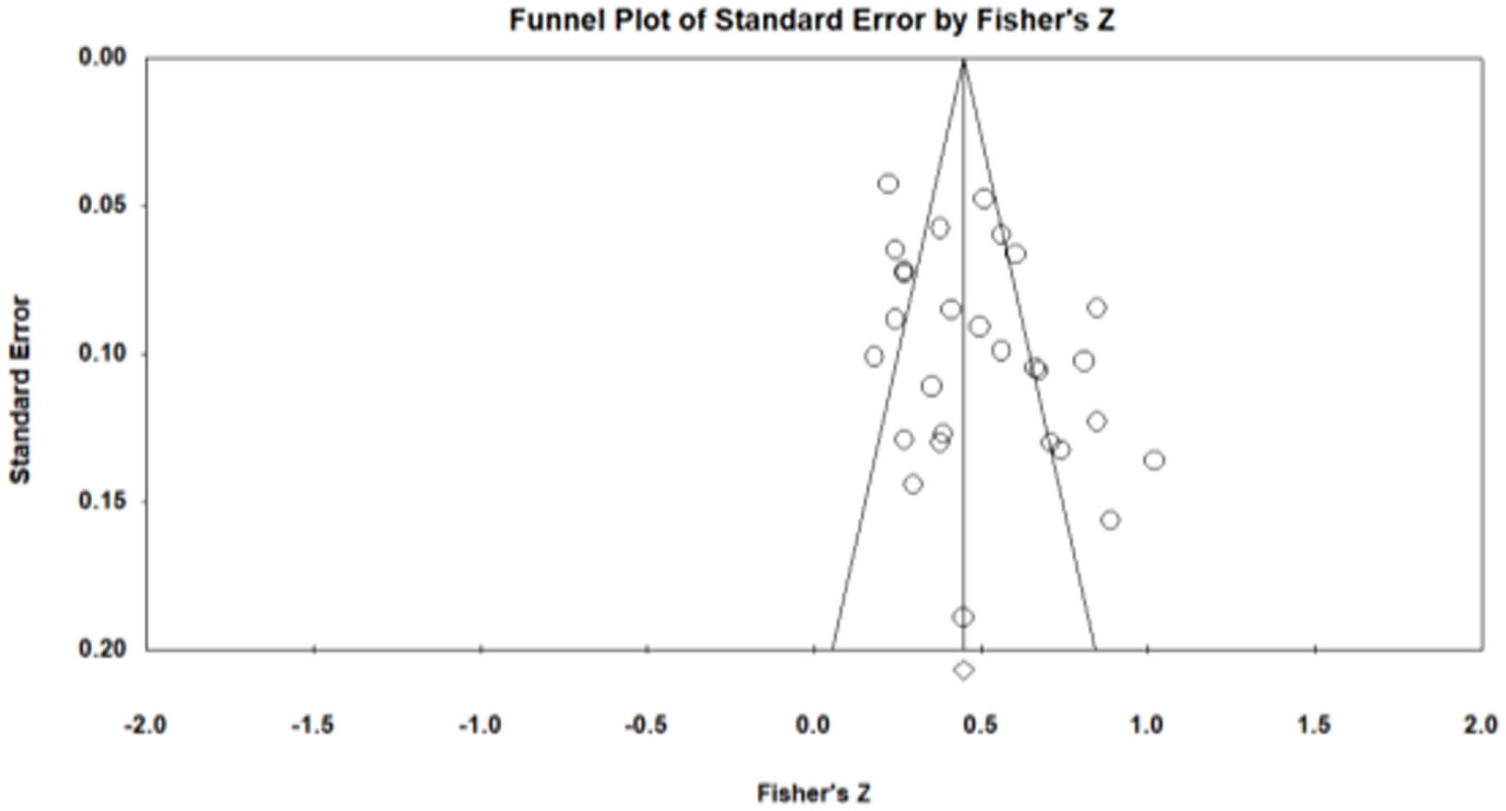
Funnel plot. Working memory—Mathematics.

**Figure 5:**
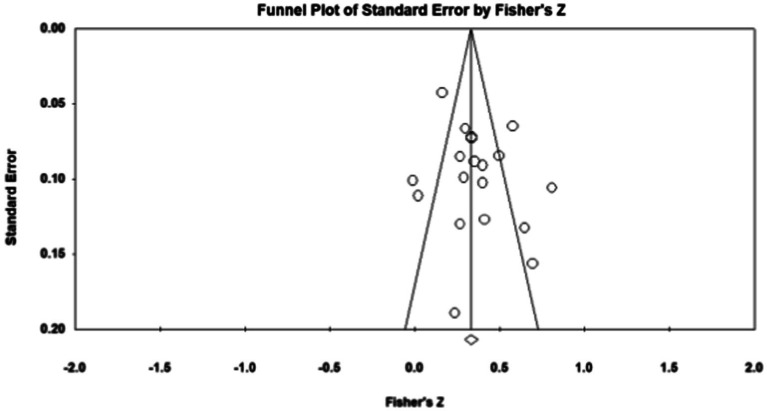
Funnel plot. Cognitive flexibility—Mathematics.

Publication bias refers to the fact that statistically significant findings are more likely to be published than non-statistically significant findings (Viechtbauer, 2007). To examine whether the results were affected by publication bias, first, journal papers, master, and doctoral dissertations were included in the data sources during sample screening, which controls publication bias to a certain extent. Second, the study further used Fisher’s Z-score funnel plot and Egger’s test for publication bias analysis. As shown in Figures 1–5, most of the effect values between executive functions and the association between each subcomponent and mathematical association are concentrated in the middle and upper part of the funnel part, and close to the average effect value, which indicates that the possibility of publication bias is small. In addition, if the *p* - value of Egger’s test is less than 0.05, there is publication bias (Egger, 1997). The test results show *p_1_* = 0.311; *p_2_* = 0.098; *p_3_* = 0.075; and *p_4_* = 0.188, so all *p* - values are greater than 0.05. This indicates that there was no obvious publication bias in the study, and the statistical data results are genuine and reliable.

### Heterogeneity analysis

Due to differences in research methods, objects, tools, evaluation criteria, etc., there may be heterogeneity in research. Therefore, a test for heterogeneity is required to determine the appropriate effect model for the study. The heterogeneity test generally uses the I^2^ test statistic method to judge the heterogeneity (Higgins et al., 2003). When I^2^ < 75%, a fixed effect model is used. In case of a high degree of heterogeneity in studies, a random effects model will be used. According to the test results, the I^2^ values were all greater than 75% (*p* < 0.001), indicating that the effect values selected in the study were all heterogeneous, so a random effect model was used for further analyses.

### Main effects test

We identified 65 effect sizes from 23 independent studies involving a total of 2,370 children from China, of whom 55.6% are female. The main effect results of the meta-analysis are shown in Table 2. A total of 18 effect values are involved between the overall executive functions and mathematical competence, with the sample size being 2,365, and the total effect size is 0.49 (95% CI [0.394, 0.585]). It can be seen that there is a significant correlation between overall executive functions and mathematical competence. Among them, the total effect size between inhibition control and mathematical competence is 0.347 (95% CI [0.221, 0.462]), involving a total of 17 effect values and a total sample size of 2,027; there are 17 effects between working memory and mathematical competence. The sample size is 2,070, and the total effect size is 0.432 (95% CI [0.318, 0.533]); the effect size between cognitive flexibility and mathematical competence is 0.370 (95% CI [0.270, 0.460]), involving 1,781 samples with 14 effect sizes. The result pointed out that there is a significant positive correlation between inhibition control, working memory and cognitive flexibility, and mathematical competence, but there are differences in the correlation between each sub-component of executive functions and mathematical competence. Specifically, R (Working memory) > R (Cognitive flexibility) > R (Inhibition control), the correlation between working memory and mathematical competence is the strongest, followed by the correlation between cognitive flexibility and mathematical competence, and the correlation between inhibition control and mathematical competence is the weakest. In addition, we performed a heterogeneity test for each group and found that there are large differences within each group, suggesting that there might be a moderating effect at play.

**Table 2:** Main effects results for executive functions and mathematical competence.

	*N*	*K*	*R*	95% CI		Homogeneity test		
				Lower	Upper	*Q*-value	Value of *p*	*I* ^2^
EFs and MS	2,365	18	0.496	0.394	0.585	151.989	0.000	88.815
IN and MS	2,027	17	0.347	0.221	0.462	149.799	0.000	89.319
UP and MS	2,070	17	0.432	0.318	0.533	143.069	0.000	88.817
CF and MS	1,781	14	0.370	0.272	0.460	67.003	0.000	80.598

N, total sample size; K, number of effect sizes; R, effect size; 95% CI, 95% confidence interval.

## Moderating effect test

### Gender

There are some differences in the association between executive functions and mathematical competence in preschool children of different genders. As shown in Table 3, from the overall perspective of executive functions, the effect size between executive functions and mathematical competence of female children is R = 0.530 (95% CI [0.470, 0.586]), and that of male children is 0.484 (95% CI [0.416, 0.548]), R_female_ > R_male_, and at the same time, the difference between groups is significant (*Q* = 74.820, *p* < 0.001, and I^2^ = 77.279). In terms of inhibition control, the effect size of between executive functions and mathematical competence of female preschool children is greater than that of male preschoolers R_female_ = 0.391 (95% CI [0.311, 0.465]) > R_male_ = 0.338 (95% CI [0.261, 0.411]). There is a significant difference between groups (*Q* = 67.278, *p* < 0.001, and I^2^ = 76.218); in terms of working memory, the effect size between male preschool children’s executive functions and mathematical competence is greater than that of female preschoolers R_male_ = 0.462 (95% CI [0.398], 0.522]) > R _female_ = 0.385 (95% CI [0.305,0.459]). The difference between groups is significant (*Q* = 81.711, *p* < 0.001, and I^2^ = 80.419); in terms of cognitive flexibility, the effect size of the association between executive functions and mathematical is greater in male than female R_male_ = 0.369 (95% CI [0.288, 0.445]) > R_female_ = 0.335 (95% CI [0.245, 0.419]). The difference between groups is significant (*Q* = 33.587, *p* < 0.001, and I^2^ = 61.295). In conclusion, the results of the heterogeneity test between groups showed significant differences between each group, and therefore, gender played a moderating role in the association between executive functions and mathematical competence in preschool children.

**Table 3:** The moderating effect of gender on executive functions and mathematical competence in preschool children.

	Gender	*N*	*K*	*R*	95% CI		Heterogeneity test within each group		
					Lower	Upper	*Q*	*P*	*I* ^2^
EFs	Male	612	9	0.484	0.416	0.548	74.820	0.000	77.279
	Female	544	9	0.530	0.470	0.586			
IN	Male	560	8	0.338	0.261	0.411	67.278	0.000	76.218
	Female	492	9	0.391	0.311	0.465			
UP	Male	647	8	0.462	0.398	0.522	81.711	0.000	80.419
	Female	495	9	0.385	0.305	0.459			
*CF*	Male	512	7	0.369	0.288	0.445	33.587	0.000	61.295
	Female	418	7	0.335	0.245	0.419			

N, sample size; K, number of effect sizes; R, effect size; 95% CI = 95% confidence interval.

### Age

The age of the subjects is further divided into three groups: 3–4 years old, 4–5 years old, and 5–6 years old, and their moderating effects on the association between executive functions and mathematical competence of preschool children were discussed, respectively. The analysis results are shown in Table 4. First, in terms of the overall link between executive functions and mathematical competence, the effect sizes varied among children of different ages, while this difference was significant (Q = 74.820, *p* < 0.001, and I^2^ = 77.279). Second, in terms of inhibition control, the effect sizes between executive functions and mathematical competence differed across age groups. For example, the effect size was 0.370 (95% CI [0.266, 0.466]) between 3 and 4 year olds, 0.424 (95% CI [0.339, 0.502]) for 4–5 year olds, and 0.279 (95% CI [0.177, 0.376]) for 5–6 year olds, and the difference between them was significant (Q = 67.278, *p* < 0.001, and I^2^ = 76.218). Similarly, there were significant differences in the association between executive functions and mathematical competence in children of different ages in terms of inhibitory control (Q = 87.711, *p* < 0.001, and I^2^ = 80.419) and cognitive flexibility (Q = 63.587, p < 0.001, and I^2^ = 61.295). In conclusion, the link between executive functions and mathematical competence in preschoolers is moderated by age differences.

**Table 4:** The moderating effect of age on executive functions and mathematical competence in preschool children.

	Age	*N*	*K*	*R*	95% CI		Heterogeneity test within each group		
					Lower	Upper	*Q*	*P*	I^2^
EFs	3–4 years	437	5	0.554	0.484	0.617	74.820	0.000	77.279
	4–5 years	233	6	0.485	0.384	0.575			
	5–6 years	456	7	0.477	0.401	0.546			
IN	3–4 years	296	4	0.370	0.266	0.466	67.278	0.000	76.218
	4–5 years	411	7	0.424	0.339	0.502			
	5–6 years	302	6	0.279	0.177	0.376			
UP	3–4 years	403	4	0.416	0.331	0.494	87.711	0.000	80.419
	4–5 years	364	7	0.429	0.339	0.511			
	5–6 years	375	6	0.444	0.357	0.523			
CF	3–4 years	275	3	0.335	0.224	0.437	63.587	0.000	61.295
	4–5 years	325	6	0.348	0.246	0.443			
	5–6 years	304	5	0.375	0.272	0.469			

N, sample size; K, number of effect sizes; R, effect size; 95% CI, 95% confidence interval.

### Instruments

The measurement of executive functions is mainly to measure the sub-components of executive functions, and the instruments selected in different studies vary. So, after statistics processing, several typical instruments were selected in each aspect of executive functions to explore the moderating effect of different instruments on the executive functions and mathematical competence of preschool children. The analysis results are shown in Table 5. In terms of inhibition control, the effect size of Stroop Task between executive functions and mathematical competence of preschool children is 0.361 (95% CI [0.311, 0.409]); the effect size of HTKS Task between executive functions and mathematical competence of preschool children is 0.360 (95% CI [0.297, 0.420]); and the difference between groups is significant (Q = 149.799, *p* < 0.001, and I^2^ = 89.319). In terms of working memory, the Fixed Box Task had a significant effect on the executive functions and mathematical competence of preschool children. The effect size between the two groups is 0.413 (95% CI [0.362, 0.462]); the effect size of the Digit-Span Task between preschool children’s executive functions and mathematical competence is 0.298 (95% CI [0.219, 0.374]); the effect size of Digit Backwards Task between executive functions and mathematical competence of preschool children is 0.524 (95% CI [0.453, 0.589]); and the difference between groups was significant (Q = 143.069, *p* < 0.001, and I^2^ = 88.817); In terms of cognitive flexibility, the effect size of FIST Task between executive functions and mathematical competence of preschool children is 0.392 (95% CI [0.334, 0.446]); the effect size of WCST Task between executive functions and mathematical competence of preschool children is 0.329 (95% CI [0.268, 0.387]); and the difference between groups is significant (Q = 67.003, *p* < 0.001, and I^2^ = 8.598). According to the heterogeneity test results between each group, the differences among the groups are all significant, which indicated that the instruments had a moderating effect between the executive functions and mathematical competence of preschool children.

**Table 5:** The moderating effect of instruments on executive functions and mathematical competence in preschool children.

	Instruments	*N*	*K*	*R*	95%CI		Heterogeneity test within each group		
					Lower	Upper	*Q*	*P*	*I* ^2^
IN	Stroop task	1,237	9	0.361	0.311	0.409	149.799	0.000	89.319
	HTKS task	1,167	5	0.360	0.297	0.420			
UP	Fixed box task	1,068	8	0.413	0.362	0.462	143.069	0.000	88.817
	Digit-span task	452	5	0.298	0.219	0.374			
	Digit backwards	454	4	0.524	0.453	0.589			
*CF*	FIST task	897	6	0.392	0.334	0.446	67.003	0.000	80.598
	WCST task	884	4	0.329	0.268	0.387			

N, sample size; K, number of effect sizes; R, effect size; 95% CI = 95% confidence interval.

## Discussion

The findings of this meta-analysis led us to conclusions regarding relations between executive functions and mathematical competence in Chinese cultural background. The overall research results indicated that executive functions and sub-dimensions of EFs (i.e., inhibition control, working memory, and cognitive flexibility) were positively associated with mathematical competence. The correlation coefficients for these findings were both medium. Furthermore, preschoolers’ age, gender, and instruments moderated these relations.

### The association between executive functions and mathematical competence in Chinese preschoolers

The meta-analysis results indicated that there was a moderately positive correlation between executive functions and mathematical competence in preschool children in the Chinese context, which is consistent with previous studies (Zhou et al., 2009; Zeng, 2020; Zhang, 2021). However, the study findings differed from previous literature’s conclusion (Liu, 2013; Ren, 2015). The first reason for such a difference is the indirect association between executive functions and mathematical competence. Studies have shown that learning quality plays a mediating role between executive functions and mathematical competence (Zhang, 2021). In addition, the association between the two may also be affected by factors such as family socioeconomic status, subjective well-being, and parental expectations (Deng et al., 2016). Second, the executive functions’ subcomponents play different roles. Because the association between each sub-dimension and mathematical competence is different, there is a moderately positive correlation between executive functions and mathematical competence. According to the results of the meta-analysis, the correlation between working memory and mathematical competence is the strongest among the executive functions’ subcomponents. This suggests that the association between working memory and mathematical competence is stronger than that of inhibition control and cognitive flexibility (Qin, 2017). In summary, the link between executive functions and mathematical competence of preschool children is not a simple linear association of binary logic as we may assume, and the internal mechanisms of development are more complex.

### Gender as a moderator

Preschool children’s gender plays a role in determining the level of correlation between executive functions and mathematical competence varied which is consistent with some previous research results (George and Robitzsch, 2018; Innabi and Dodeen, 2018). Here are some possible explanations: First, there are differences in the lower brain and hormones of different genders. Different brain regions are involved in the processing of mathematically complex problems between males and females, which may lead to different strategies for solving complex problems (Zhou and Chen, 2011). In addition, boys and girls secrete different levels of hormones that may affect children’s visual space ability (Liu and Tao, 2012). Second, males and females differ in the cognitive mechanism of mathematical concepts, with males being generally functional while females tend to be descriptive. Males are better at exploring certain tools playfully, and at the same time have a plan, while girls have strong internal positive behaviors, and are accustomed to the division of labor and cooperative learning methods. It is precise because of the different cognitive mechanisms of mathematical concepts that there are significant differences between different genders (Inge Schwank, 1996). In addition, different genders may be endowed with different cognitive styles and expression abilities (Skogli et al., 2013).

### Age as a moderator

Another factor—children’s age would also impact how correlated they are between executive functions and mathematical competence. The main reason is that the development of executive functions is staged, and the influence of executive functions of preschool children of different ages on children’s mathematical competence is different. Specifically, inhibition control is the ability to consciously suppress dominant responses in the first place, and this ability increases with age. Studies have shown that inhibition control reaches a stage of rapid development when children are 4 years old (Frye et al., 1995). Second, working memory is the core part of the executive functions of preschool children, and it is the key mechanism of children’s mathematics learning. Working memory plays a huge role, especially in preschool children at a younger age (Wang, 2019). Third, children’s cognitive flexibility plays different roles at different ages. Studies have shown that children’s cognitive flexibility begins to develop at the age of 3 years, and the age of 4–5 is a period of rapid development of this ability (Fei et al., 2020). Therefore, because the development of executive functions in children’s preschool stage has a large difference, there is a significant difference in the association with mathematical competence.

### Instruments as a moderator

According to the results of the meta-analysis, there are significant differences in the association between executive functions and mathematical competence of preschool children under different instruments. The main reason is that the content of the test tasks is quite different. For instance, the Stroop Task, which tests children’s inhibition control ability, let children quickly say “night” when they see a picture of the “sun” and say “day” when they see a picture of the “moon.” Also, a blue-red task that examines inhibition control has children say “blue” when they see red, and “red” when they see blue. Although there is not much difference between the two instruments, they are quite different in content. Children’s cognition of the objective objects “sun” and “moon” is different from the cognition of “red” and “blue” colors, and there are also great differences in essence. Therefore, there are significant differences in the results measured by different instruments.

### Limitations and future directions

There are several limitations to this study. Firstly, the effect mechanism of preschool children’s executive functions on mathematical competence needs further clarification. At present, it is uncertain how executive functions affect mathematical competence, and the issue in terms of how the subcomponents of executive functions are related also needs further studies. As some studies have shown, there may be other factors (such as children’s language, vocabulary, spatial ability, etc.) at play between executive functions and mathematical competence. Therefore, issues such as how executive functions (including each sub-component) affect children’s mathematical competence, what is the association between the sub-components, and whether there is some interaction mechanism need further research. Secondly, the demonstration of the causal relationship between the two in the existing research is insufficient. Future study should deepen the discussion on this aspect to further prove the existence of the causal association between the two through experimental research and longitudinal research. Finally, our sample only considered preschool children between 3 and 7 under the Chinese cultural background, and thus did not include older children. Future research should expand the sample group to verify the validity of research conclusions from a broader sample group.

## Conclusion

This study used meta-analysis research methods to explore the real association between executive functions and mathematical competence in preschool children in the Chinese context. The research results showed that there was a moderately positive correlation between the executive functions and mathematical competence of Chinese preschool children. Among them, the association between working memory and mathematical competence was the strongest. In addition, the association was also moderated by gender, age, and instruments. Our findings have clarified the debate on existing research viewpoints, illustrated the real association between preschool children’s executive functions (including each sub-component) and mathematical competence, and provided a research basis and reference for subsequent scientific research.

## Data availability statement

The original contributions presented in the study are included in the article/supplementary material, further inquiries can be directed to the corresponding author.

## Author contributions

ZZ: conceptualization, methodology, writing—original draft, and review and editing. YX: conceptualization, formal analysis, and writing—review and editing. RJ, MZ, and HZ: writing—review and editing. CY: writing—review and editing, and supervision. All authors contributed to the article and approved the submitted version.

## Funding

This work was supported by the National Ethnic Affairs Commission of the People’s Republic of China “Research on Early Childhood Education and Development in Minority Areas (2021-GMI-010) Ethnic Research Project.”

## Conflict of interest

The authors declare that the research was conducted in the absence of any commercial or financial relationships that could be construed as a potential conflict of interest.

## Publisher’s note

All claims expressed in this article are solely those of the authors and do not necessarily represent those of their affiliated organizations, or those of the publisher, the editors and the reviewers. Any product that may be evaluated in this article, or claim that may be made by its manufacturer, is not guaranteed or endorsed by the publisher.
